# Supplementary material for: Genome-Wide Identification of the NAC Transcription Factors in Gossypium hirsutum and Analysis of Their Responses to Verticillium wilt
Source: Plants (Basel). 2022 Oct 10;11(19):2661. doi: 10.3390/plants11192661 (PMC9571985; doi:10.3390/plants11192661)
Supplement: Supplementary file 1 [file plants-11-02661-s001.zip › figure S1-S3.pdf]

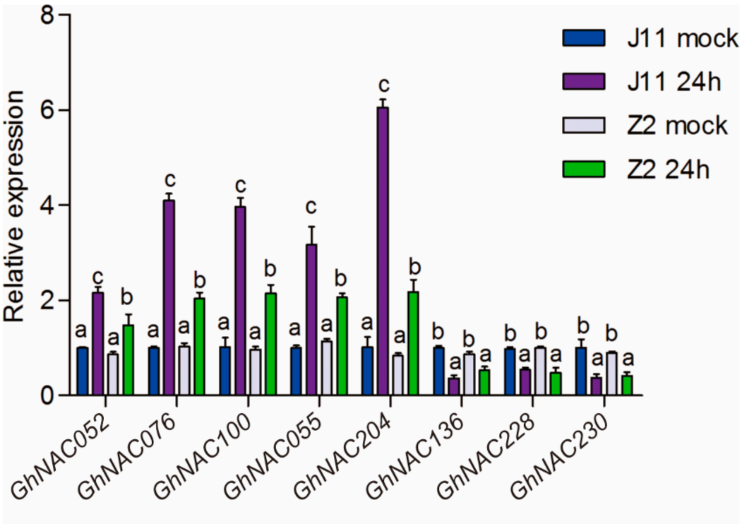

Figure S1. qRT-PCR validation of selected *GhNACs*. *GhUBQ7* is used as an internal reference gene and data are means ± SD from three biological replicates. Different letters indicate significant differences, $p < 0.05$ (ANOVA test).

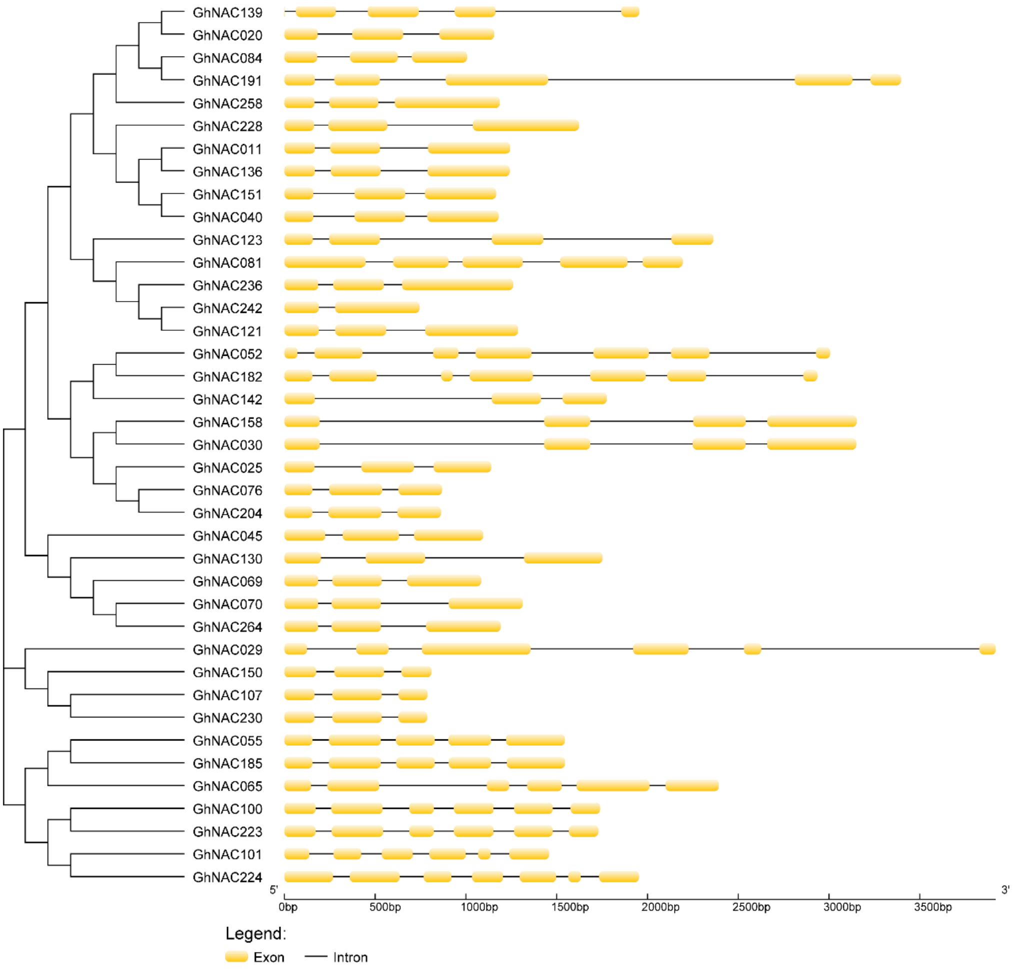

Figure S2. Exon-intron structure analysis of DEGs in putative *GhNACs*. Phylogenetic tree of DEGs constructed with MEGA 7.0 by the NJ method.

Bootstrap values from 1000 replicates are indicated at each branch.

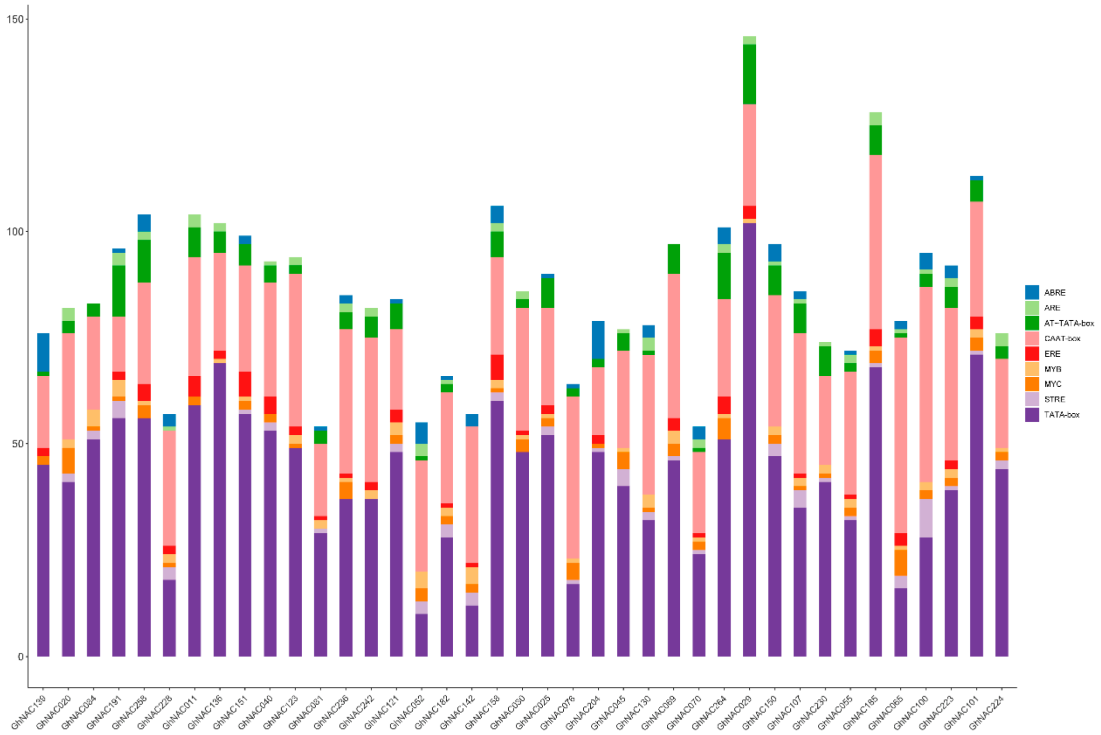

Figure S3. cis-Element analysis of DEGs in putative *GhNACs* promoters. Different cis-elements with the same or similar functions are shown in the same color.
