# Supplementary material for: Genome-Wide Identification of the NAC Transcription Factors in Gossypium hirsutum and Analysis of Their Responses to Verticillium wilt
Source: Plants (Basel). 2022 Oct 10;11(19):2661. doi: 10.3390/plants11192661 (PMC9571985; doi:10.3390/plants11192661)
Supplement: Supplementary file 1 [file plants-11-02661-s001.zip › Table S3 Summary of transcriptome sequencing results after filtering.docx]

**Supplementary Table S3. Summary of transcriptome sequencing results after filtering**

| **Sample** | **Clean reads Mapping rate (%)** | **Q30 (%)** |
| --- | --- | --- |
| J11 mock-1 | 18,919,087 89.34 | 92.43 |
| J11 mock-2 | 18,613,300 89.37 | 93.62 |
| J11 mock-3 | 19,327,874 88.90 | 92.41 |
| J11 24h-1 | 20,125,921 74.01 | 92.56 |
| J11 24h-2 | 20,901,469 79.65 | 92.59 |
| J11 24h-3 | 21,810,013 73.62 | 92.11 |
| Z2 mock-1 | 20,137,350 94.79 | 92.27 |
| Z2 mock-2 | 22,658,917 93.43 | 92.28 |
| Z2 mock-3 | 21,816,358 95.20 | 92.38 |
| Z2 24h-1 | 24,645,435 77.93 | 92.64 |
| Z2 24h-2 | 24,087,659 76.05 | 92.37 |
| Z2 24h-3 | 21,823,327 79.84 | 92.41 |
