# Supplementary figures and images for: Genome-Wide Identification of the NAC Transcription Factors in Gossypium hirsutum and Analysis of Their Responses to Verticillium wilt
Source: Plants (Basel). 2022 Oct 10;11(19):2661. doi: 10.3390/plants11192661 (PMC9571985; doi:10.3390/plants11192661)

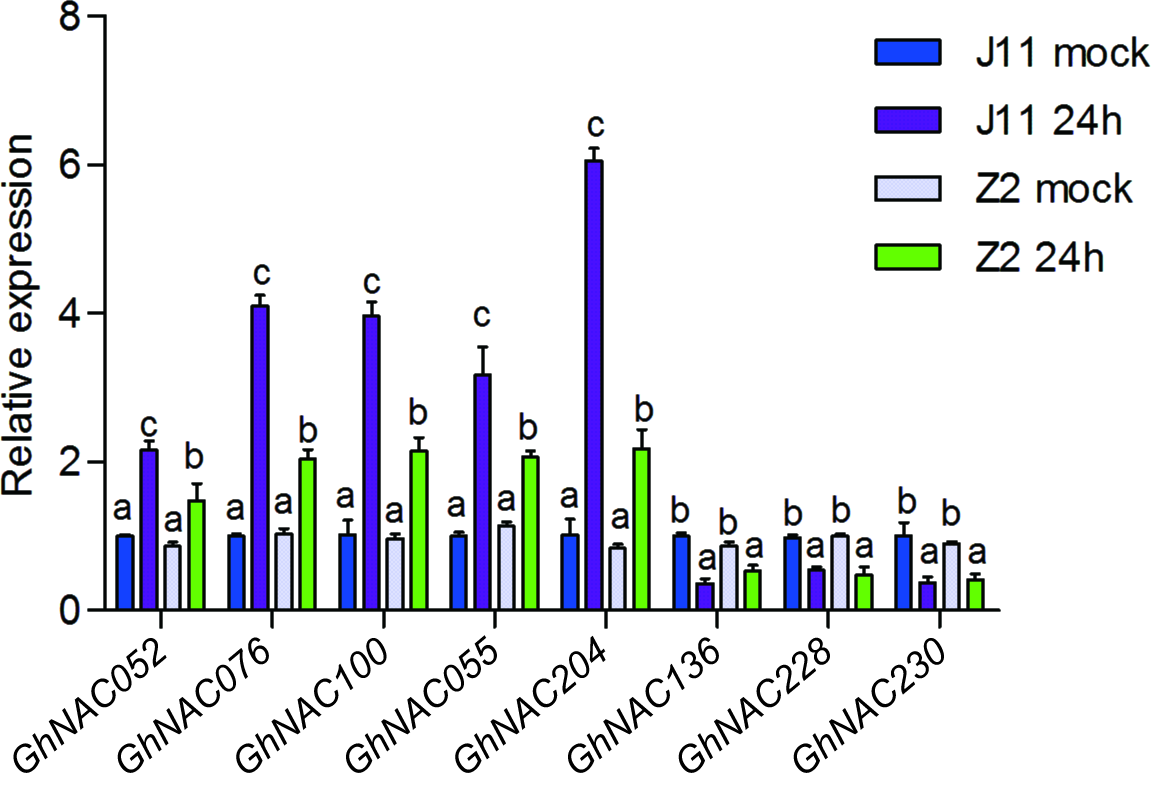

Supplement: Supplementary file 1 [file plants-11-02661-s001.zip › fig.S1 qRT-PCR validation of selected GhNACs.tif]

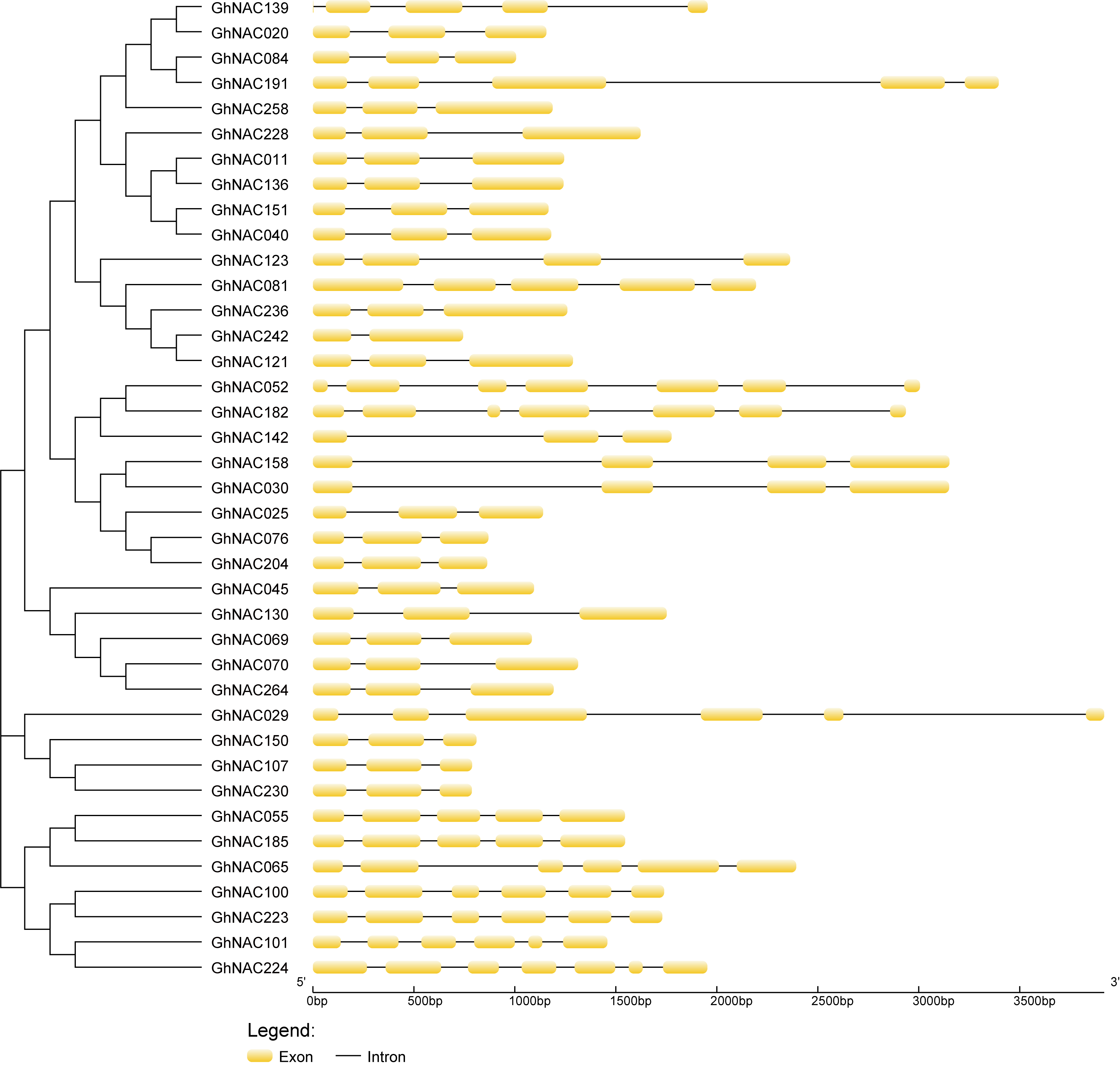

Supplement: Supplementary file 1 [file plants-11-02661-s001.zip › figS2 Exon-intron structure analysis of DEGs in putative GhNACs.tif]

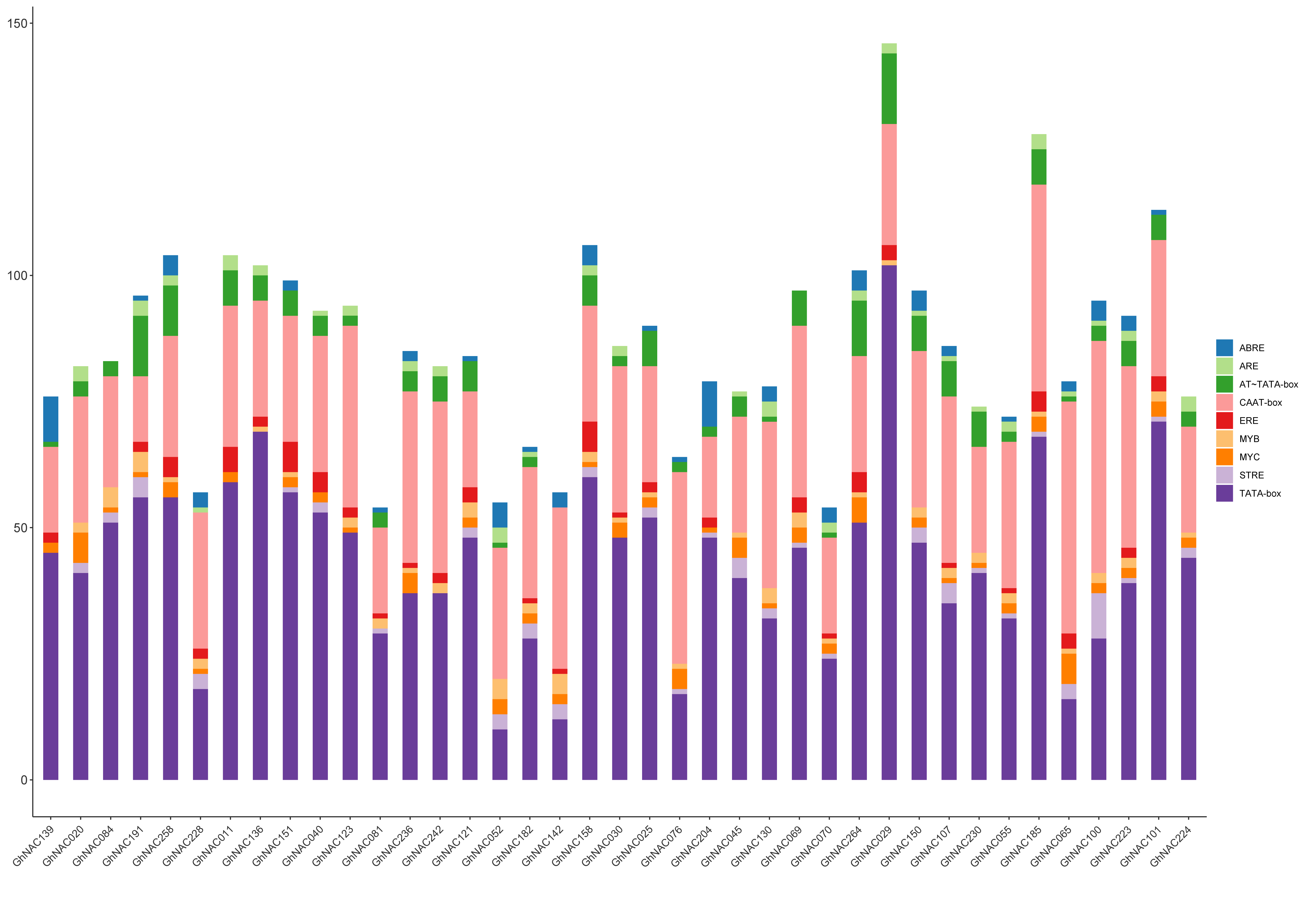

Supplement: Supplementary file 1 [file plants-11-02661-s001.zip › figS3 cis-Element analysis of DEGs in putative GhNACs promoters.tif]
